# The Interactive Effects of Sedentary Time, Physical Activity, and Fat Mass on Insulin Resistance in the Young Population

**DOI:** 10.1210/clinem/dgae135

**Published:** 2024-03-05

**Authors:** Andrew O Agbaje

**Affiliations:** Institute of Public Health and Clinical Nutrition, School of Medicine, Faculty of Health Sciences, University of Eastern Finland, 70211 Kuopio, Finland; Children's Health and Exercise Research Centre, Department of Public Health and Sports Sciences, Faculty of Health and Life Sciences, University of Exeter, Exeter EX1 2LU, UK

**Keywords:** pediatrics, type 2 diabetes, causality, longitudinal study, childhood obesity, mediation

## Abstract

**Context:**

Recent evidence in 9-year-old children with overweight/obesity followed up for 7 years until late adolescence concluded that increased physical activity (PA) decreased the risk of high fasting glucose, low insulin sensitivity, and secretion. However, whether this effect persists until young adulthood is unknown.

**Objective:**

This observational study examined the effects of cumulative sedentary time (ST), light PA (LPA), and moderate to vigorous (MVPA) on glucose, insulin, and homeostatic model assessment for insulin resistance (HOMA-IR) in 11-year-old children followed up for 13 years until young adulthood.

**Methods:**

Altogether 792 children from the Avon Longitudinal Study of Parents and Children, UK, who had data on at least 2 measures of accelerometer-based movement behaviour during 11-, 15-, and 24-year follow-up clinic visits with complete fasting glucose, insulin, and HOMA-IR measures at ages 15, 17, and 24 years were included. ST, LPA, and MVPA were measured with an accelerometer.

**Results:**

Cumulative ST from ages 11-24 years was associated with increased odds (odds ratio 1.20, 95% CI 1.00-1.44, *P* = .047) and cumulative LPA was associated with the decreased odds of hyperinsulinemia (0.80, 0.66-0.96, *P* = .017) among participants with overweight/obesity. Cumulative MVPA was inversely associated with insulin but after accounting for the mediating role of fat mass, MVPA effect on lowering insulin decreased by 58% resulting in statistical nonsignificance. In the temporal path analyses, among participants with overweight/obesity, higher glucose at age 15 years was associated with lower LPA and MVPA at 24 years. Higher LPA at 15 years was associated with lower insulin and HOMA-IR at 24 years and vice versa.

**Conclusion:**

Promoting LPA while decreasing body fat mass and ST may be considered crucial intervention targets to attenuate the risk of hyperinsulinemia and insulin resistance from childhood through young adulthood.

Long-term complications of youth-onset type 2 diabetes have been reported at age 24 years in children diagnosed with diabetes at age 11 years, warranting a global urgency for the prevention of the precursors of this disease—dysglycemia, hyperinsulinemia, and insulin resistance (1). Recent physical activity (PA) guidelines recommended decreasing sedentary time (ST) and accruing at least 60 minutes/day of moderate to vigorous PA (MVPA) in children and adolescents less than 18 years for the prevention of cardiometabolic diseases (2). In 9-year-old children who were mostly overweight and obese and followed up for 2 years, higher MVPA and lower ST improved insulin sensitivity through their effect on adiposity (3). Similarly, MVPA attenuated homeostatic model assessment for insulin resistance (HOMA-IR) during midadolescence in 9-year-old children followed up for 6 years (4). Higher MVPA and less ST potentially improved insulin sensitivity and reduced insulin secretion in 9-year-old children with overweight and obesity followed-up for 7 years, until late adolescence (5). Since high total body fat and abdominal fat increased the risk of elevated insulin resistance by 12% to 21% in the young population, the role of movement behavior in interrupting the fat mass–insulin resistance vicious cycle in late adolescence is of clinical and public health importance (6).

A higher glucose level may temporally precede higher ST and lower PA in children and adolescents diagnosed with type 1 and 2 diabetes, but it remains unknown whether higher glucose levels may precede lower MVPA in children and adolescents with healthy weight or overweight/obese (7-9). Identified knowledge gaps in the pediatric population include the lack of childhood through young adulthood longitudinal relationships of ST and MVPA, especially light PA (LPA) on progressive changes in glycemia, insulin, and HOMA-IR (1-3, 5, 10, 11). Cumulative LPA from childhood was recently shown to be more effective than MVPA in decreasing inflammation, fat mass, and cholesterol levels during growth from childhood to young adulthood (12-14). It remains unclear whether movement behaviors may potentially influence dysglycemia and HOMA-IR during growth from childhood through young adulthood via the mediating path of increased body fat mass, muscle mass, lipids, and inflammation, or due to reverse causation bias (10-14). Clarifying potential temporal inter-relations of movement behaviors with metabolic indices is an important inquiry that has implications for mounting effective childhood metabolic disease prevention programs (1, 2, 5, 10-13).

The present observational study (1) examined the longitudinal associations of accelerometer-based repeatedly measured ST, LPA, and MVPA, with repeated measures of fasting glucose, insulin, and HOMA-IR in 11-year-old children followed up for 13 years in the total cohort, participants with overweight/obesity, and according to sex; (2) assessed the extent to which the associations of movement behaviors with glucose, insulin, and HOMA-IR was mediated by fat mass, muscle mass, lipid, and inflammation; and (3) examined the temporal interrelations among activity levels and glucose, insulin, and HOMA-IR in the total cohort and participants with overweight/obesity using data from the Avon Longitudinal Study of Parents and Children (ALSPAC) birth cohort, England, UK.

## Materials and Methods

### Study Cohort

Data were from the ALSPAC birth cohort, which investigates factors that influence childhood development and growth. Pregnant women resident in Avon, UK, with expected dates of delivery between April 1 1991 and December 31 1992 were invited to take part in the study. In total, 20 248 pregnancies have been identified as being eligible and the initial number of pregnancies enrolled was 14 541. Of the initial pregnancies, there was a total of 14 676 fetuses, resulting in 14 062 live births and 13 988 children who were alive at 1 year of age. When the oldest children were approximately 7 years of age, an attempt was made to bolster the initial sample with eligible cases who had failed to join the study originally. As a result, when considering variables collected from the age of 7 onwards (and potentially abstracted from obstetric notes) there are data available for more than the 14 541 pregnancies mentioned above. The number of new pregnancies not in the initial sample (known as Phase I enrollment) that are currently represented in the released data and reflecting enrollment status at the age of 24 is 906, resulting in an additional 913 children being enrolled (456, 262, and 195 recruited during Phases II, III, and IV respectively). The total sample size for analyses using any data collected after the age of 7 is therefore 15 447 pregnancies, resulting in 15 658 fetuses. Of these, 14 901 children were alive at 1 year of age. Regular clinic visits of the children commenced at 7 years of age and are still ongoing into adulthood. Study data at 24 years of age were collected and managed using REDCap electronic data capture tools (15). Details of participant selection are presented in the flow chart (Fig. S1 (16)). In this study, 792 participants with at least 2 timepoints valid ST, LPA, and MVPA measurements at either age 11-, 15-, or 24-year clinic visit and with complete glucose, insulin, and HOMA-IR measures at 15-, 17-, and 24-year clinic visits were eligible for analyses (Fig. S1 (16)). The excluded participants who had 1 or no timepoint measure of ST and PA during the 13-year-long follow-up study had baseline characteristics similar to those included in the study (Table S1 (16)). Ethics approval for the study was obtained from the ALSPAC Ethics and Law Committee and the Local Research Ethics Committees. Informed consent for the use of data collected via questionnaires and clinics was obtained from participants following the recommendations of the ALSPAC Ethics and Law Committee at the time (17-19). Consent for biological samples has been collected in accordance with the Human Tissue Act (2004). Please note that the study website contains details of all the data that are available through a fully searchable data dictionary and variable search tool (http://www.bristol.ac.uk/alspac/researchers/our-data/).

### Exposures: Sedentary Time and Physical Activity Assessment

ST, LPA, and MVPA were assessed with an ActiGraphTM (LLC, Fort Walton Beach, FL, USA) accelerometer worn on the waist for 7 consecutive days at 11- and 15-year clinic visits whereas at 24 years movement behavior was assessed using ActiGraph GT3X + accelerometer device worn for 4 consecutive days. There is a strong absolute agreement between the Actigraph models (intraclass correlation coefficient 0.99, 95% CI 0.98-0.99) thus making it acceptable to use different models within a study (20). A valid day was defined as providing data for at least 10 hours per day (excluding sequences of 10 or more minutes with consecutive 0 counts) and children were only included in the analyses if they provided at least 3 valid days of recording (21). The devices capture movement in terms of acceleration as a combined function of frequency and intensity. Data are recorded as counts that result from summing postfiltered accelerometer values (raw data at 30 Hz) into 60-second epoch units. Data were processed using Kinesoft software, version 3.3.75 (Kinesoft), according to the established protocol (22, 23). Activity counts per minute thresholds validated in young people were used to calculate the amount of time spent: MVPA, >2296 counts per minute (cpm); for LPA, 100 to 2296 cpm; and for ST, 0 to <100 cpm at ages 11 and 15 years, but 2020 cpm for the 24-year MVPA assessment (22, 24, 25). MVPA was classified according to PA guideline recommendations of <40 minutes/day as low (reference), 40 to <60 minutes/day as moderate, and ≥60 minutes/day as high (2). The 40 to <60 minutes/day of MVPA was based on the lowest tertile cutpoint (39.95 minutes) for MVPA in the total population. The cut point between the middle and highest MVPA tertile was 59.8 minutes/day in line with the current PA guideline (2).

### Outcomes: Fasting Glucose, Insulin, and Insulin Resistance

There were no measures of fasting insulin and glucose at age 11 years. Fasting glucose and insulin were assessed at age 15-, 17-, and 24-year clinic visits. Fasting glucose was measured using standard protocols. Fasting insulin was assessed using an ultrasensitive automated microparticle enzyme-linked immunosorbent assay (Mercodia, DSL, London, UK), ALSPAC (RRID:SCR_007260), which does not cross-react with proinsulin (26); the sensitivity of the immunoassay was 0.07 mU/L. We calculated HOMA-IR from (fasting plasma insulin × fasting plasma glucose/22.5) (27). Hyperglycemia was categorized as fasting glucose >6.1 mmol/L, hyperinsulinemia was categorized as fasting insulin >11.28 mU/L (28, 29). HOMA-IR >75th percentile was categorized as high.

### Covariates: Anthropometry and Body Composition

Anthropometry (height and weight) of participants at ages 11, 15, and 24 years were assessed in line with standard protocols, and body mass index (BMI) was computed as weight in kilograms per height in meters squared (25, 30). BMI was categorized as healthy weight if <25 kg/m^2^ and overweight/obese if ≥25 kg/m^2^. Body composition (total fat mass, trunk fat mass, and lean mass) was assessed using a dual-energy X-ray absorptiometry scanner at the 11-, 15-, and 24-year clinic visits as previously described (13, 25, 28, 30-32).

### Covariates: Cardiometabolic, Socioeconomic, and Lifestyle Factors

Heart rate and systolic and diastolic blood pressure were measured with Omron monitor at ages 11, 15, and 24 years as previously detailed (25, 30). Using standard protocols, fasting blood samples at ages 15, 17, and 24 years were collected, spun, and frozen at −80 °C, and a detailed assessment of fasting high-sensitivity C-reactive protein, low-density lipoprotein cholesterol, high-density lipoprotein cholesterol, and triglycerides have been reported (coefficient of variation was <5%) (25, 28, 30, 33, 34). At the 17-year clinic visit, participants were briefly asked about their personal and family (mother, father, and siblings) medical history such as a history of hypertension, diabetes, high cholesterol, and vascular disease. All participants had attained puberty at the 17-year clinic visit using a time (years) to age at peak height velocity objective assessment derived from superimposition by translation and rotation mixed-effects growth curve analysis (25, 35). The participant's mother's socioeconomic status collected during childhood clinic visit was grouped according to the 1991 British Office of Population and Census Statistics classification (36). Questionnaires to assess smoking behavior were administered at the 13-, 15-, and 24-year clinic visits. A specific question regarding whether participants smoked in the last 30 days was used as an indicator of current smoking status.

### Statistical Analysis

Cohort descriptive characteristics were summarized as means and SD, medians, and interquartile ranges, or frequencies and percentages. We explored sex differences using independent t-tests, Mann–Whitney U tests, or chi-square tests for normally distributed, skewed, or dichotomous variables, respectively. Multicategory variables were analyzed using a 1-way analysis of variance. Normality was assessed by histogram curve, quantile–quantile plot, and Kolmogorov–Smirnov tests. We conducted a logarithmic transformation of skewed variables and confirmed normality prior to further analysis.

#### Mediation path analyses

Mediating path analyses using structural equation models separately examined the mediating role of either cumulative total fat mass or lean mass on the longitudinal associations of cumulative ST, LPA, or MVPA with each of cumulative glucose, insulin, and HOMA-IR. Analyses were adjusted for age, sex, high-density lipoprotein cholesterol, low-density lipoprotein cholesterol, triglyceride, high-sensitivity C-reactive protein, family history of hypertension and cardiovascular diseases, smoking status, socioeconomic status, heart rate, total fat mass, lean mass, ST, LPA, MVPA, and glucose or insulin depending on the mediator, predictor, or outcome. The path models had 3 equations per regression analysis: the longitudinal associations of cumulative ST, LPA, or MVPA with cumulative total fat mass or lean mass (Equation 1); the longitudinal associations of cumulative total fat mass or lean mass with glucose, insulin or HOMA-IR (Equation 2); and the longitudinal associations of cumulative ST, LPA, and MVPA with cumulative glucose, insulin or HOMA-IR (Equation 3, total effect), and Equation 3′ (direct effect) accounted for the mediating role of total fat mass or lean mass on the longitudinal associations of cumulative ST, LPA, and MVPA with cumulative glucose, insulin or HOMA-IR. The proportion of mediating or suppressing roles was estimated as the ratio of the difference between Equation 3 and Equation 3′ or the multiplication of Equations 1 and 2 divided by Equation 3 and expressed in percentage. A mediating or indirect role is confirmed when there are statistically significant associations between (1) the predictor and mediator, (2) the predictor and outcome, (3) the mediator and outcome, and when (4) the longitudinal associations between the predictor and outcome variable were attenuated upon inclusion of the mediator (37, 38). However, when the magnitude of the longitudinal association between the predictor and outcome is increased upon the inclusion of a third variable, a suppression is confirmed (37). We additionally examined the mediating or suppressing role of lipids and inflammatory marker on the longitudinal associations of cumulative ST, LPA, and MVPA with cumulative glucose, insulin, or HOMA-IR. Path analyses were conducted with 1000 bootstrapped samples.

#### Temporal causal path analyses

Lastly, structural equation modeling with autoregressive cross-lagged design was employed to examine the separate temporal associations of ST, LPA, and MVPA with each of glucose, insulin, or HOMA-IR at ages 15 and 24 years only, due to the lack of fasting metabolic data at age 11 years. The cross-lagged models first tested the separate associations of ST, LPA, and MVPA at 15 years with each of glucose, insulin, or HOMA-IR at 24 years. Next, the separate associations of glucose, insulin, or HOMA-IR at 15 years with ST, LPA, and MVPA at 24 years were examined. These models were adjusted for all covariates measured at 15 years. In the cross-lagged design, the potential association could be ST, LPA, and MVPA leading to metabolic risks, metabolic risks leading to ST, LPA, and MVPA, or bidirectional associations of ST, LPA, and MVPA with metabolic risks. If a path from ST, LPA, and MVPA at time t-1 (15 years) to each of glucose, insulin or HOMA-IR at time t-2 (24 years) reach statistical significance (*P* < .05), changes in the earlier variables are considered to lead to changes in the later, and vice versa. A stronger predictive effect is determined by a larger standardized regression coefficient. We concluded that the cross-lagged models had good fits with the following indices: the root mean square error of approximation <0.05, the normed fit index, relative fit index, incremental fit index, Tucker–Lewis fit index, comparative fit index, >0.90 for all (39). Error terms were included in the cross-lagged model. The cross-lagged path analysis was also conducted for participants who were categorized as overweight and obese at age 24 years follow-up. The plausible relationships between exposures, outcomes, and confounders are portrayed in the directed acyclic graph shown elsewhere (Fig. S2 (16)).

#### Analyses of longitudinal associations

We examined the separate longitudinal associations of each of the 13-year ST, LPA, and MVPA progression (11-24 years) with each of glucose, insulin, and HOMA-IR measured at ages 15, 17, and 24 years using generalized linear mixed-effect models (GLMMs) with identity link in the total cohort and GLMMs with logit link in participants with overweight and obesity. The GLMM is robust for handling highly correlated variables such as ST and LPA. The optimal model with the lowest Bayesian information criteria was the model with sex as the main effect, a random intercept modeled for the subject to account for within-individual correlations, and an unstructured correlation matrix. We selected a random effect variance component type and determined the effect of the predictor trajectory on the progression in repeated outcome measures. While the mixed-effect model assumes that the data are missing at random and is robust for accounting for missing data at follow-up, we elected to additionally conduct 20 cycles of multiple imputations to account for missing data using Markov Chain Monte Carlo method, especially in the predictor follow-up measures. In this population, 20 cycles of multiple imputations have been shown to have a relative efficiency of over 98% in simulating real data (24, 28, 30). The GLMM accounted for baseline ST, LPA, MVPA predictors, metabolic outcomes, and covariates and their repeated measures. Model 1 was adjusted for sex, family history of hypertension/diabetes/high cholesterol/vascular disease, socioeconomic status, and other time-varying covariates measured at 3 timepoints such as age, low-density lipoprotein cholesterol, triglyceride, high sensitivity C-reactive protein, high-density lipoprotein cholesterol, heart rate, systolic blood pressure, smoking status, total fat mass, and lean mass, and glucose or insulin depending on the outcome. Model 2 was an additional adjustment for ST, LPA, or MVPA depending on the predictor to mutually adjust for activity levels. A different GLMM was conducted for each sex, and sex-based analyses were not adjusted for sex. Cumulative HOMA-IR outcomes were not adjusted for cumulative glucose and insulin.

Collinearity diagnoses were performed and accepted results with a variance inflation factor <5, considered differences and associations with a 2-sided *P* < .05 as statistically significant, and made conclusions based on effect estimates and their CI. Covariates were identified based on previous studies (2, 3, 5, 10, 28, 36, 40-43). We applied Sidak correction for potential multiple comparisons. Analyses involving 8% of a sample of 10 000 ALSPAC children at 0.8 statistical power, 0.05 alpha, and 2-sided *P* value would show a minimum detectable effect size of 0.09 SDs if they had relevant exposure for a normally distributed quantitative variable (44). All statistical analyses were performed using SPSS statistics software, Version 27.0 (IBM Corp, Armonk, NY, USA), and mediation analyses and autoregressive cross-lagged temporal causal path structural equation modeling were conducted using IBM AMOS version 27.0.

## Results

**Figure 1.**
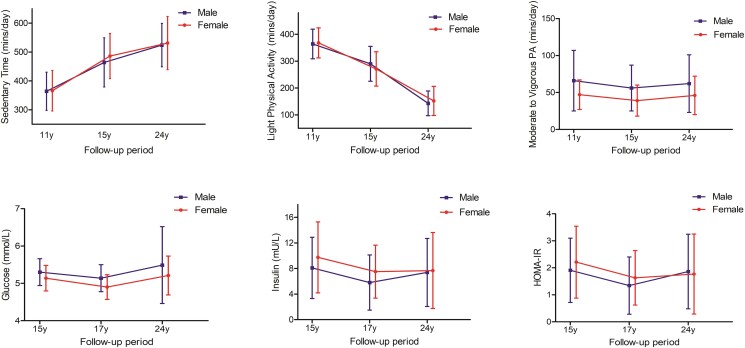
Trajectory data are presented as mean values ± SD for movement behaviors and fasting glucose while median values ± IQR for fasting insulin and homeostatic model of insulin resistance (HOMA-IR) in 792 participants: Male, n = 337; Female, n = 455.

Among 792 participants ST increased while LPA decreased from ages 11 through 24 years in both males and females (Table 1 and Fig. 1). MVPA in minutes/day had a U-shaped increase in both males and females, with males accruing more ≥60 minutes/day of MVPA across the 13-year follow-up (Table 1 and Fig. 1). From ages 15 through 24 years, females had higher insulin and HOMA-IR but lower glucose than males in a U-shaped pattern (Table 1 and Fig. 1). Other characteristics are described in Table 1 and elsewhere (Tables S1-7 (16)).

**Table 1.** Descriptive characteristics of cohort participants

Age at clinic visits/follow-up	11 years			15 years			24 years		
**Variables**	**Male (n = 337)**	**Female (n = 455)**	***P* value**	**Male (n = 337)**	**Female (n = 455)**	***P* value**	**Male (n = 337)**	**Female (n = 455)**	***P* value**
**Anthropometry**									
Age at clinic visit (years) mean (SD)	11.70 (0.20)	11.71 (0.21)	.738	15.38 (0.22)	15.39 (0.24)	.634	24.57 (0.79)	24.45 (0.77)	.033
Height (m) mean (SD)	1.51 (0.07)	1.52 (0.07)	.139	1.75 (0.07)	1.65 (0.06)	<.0001	1.80 (0.07)	1.67 (0.06)	<.0001
Weight (kg) median (IQR)	41.20 (11.9)	42.0 (12.0)	.208	63.0 (13.1)	56.80 (10.9)	<.0001	79.30 (17.32)	64.15 (16.55)	<.0001
White ethnicity, n (%)	303 (96.2)	410 (97.9)	.188	NA			NA		
**Body composition**									
Total fat mass (kg) median (IQR)	8.41 (8.40)	10.76 (7.52)	<.0001	8.59 (7.51)	16.80 (8.79)	<.0001	18.53 (11.60)	21.16 (11.12)	<.0001
Lean mass (kg) median (IQR)	29.86 (5.29)	28.69 (6.39)	<.0001	50.0 (8.59)	36.89 (4.81)	<.0001	56.90 (10.99)	40.93 (6.34)	<.0001
Body mass index (kg/m^2^) median (IQR)	18.07 (4.27)	18.15 (3.95)	.579	20.31 (3.73)	20.83 (4.08)	.009	24.38 (5.10)	23.09 (5.57)	<.0001
Overweight/obese BMI ≥25 kg/m^2^ (n,%)	21 (6.3)	17 (3.7)	.130	38 (11.3)	54 (11.9)	.823	145 (43.2)	145 (32.4)	.002
**Vascular measures**									
Heart rate (beat/mins) mean (SD)	74 (11)	78 (11)	<.0001	72 (12)	77 (11)	<.0001	65 (11)	68 (9)	<.0001
Systolic blood pressure (mmHg) mean (SD)	105 (9)	105 (10)	.891	127 (10)	120 (10)	<.0001	123 (10)	111 (9)	<.0001
Diastolic blood pressure (mmHg) mean (SD)	58 (6)	58 (6)	.919	68 (8)	66 (9)	.012	67 (8)	66 (8)	.009
**Lifestyle factors**									
Smoked in the last 30 days, n (%)	<5 (0.3)	8 (1.8)	.086	24 (7.3)	55 (12.2)	.030	82 (24.6)	105 (23.2)	.672
Family history of H-D-C-V, n (%)	94 (31.6)	118 (28.1)	.319	NA			NA		
Sedentary time (min/day) mean (SD)	364 (66)	366 (70)	.807	464 (85)	486 (78)	.001	524 (75)	531 (92)	.615
Light physical activity (min/day) mean (SD)	364 (55)	368 (56)	.317	290 (65)	271 (64)	<.0001	143 (46)	152 (54)	.246
MVPA (min/day) mean (SD)	66 (41)	47 (20)	<.0001	56 (31)	39 (21)	<.0001	62 (39)	46 (26)	.001
MVPA <40 minutes/day	54 (16.5)	175 (40)	<.0001	93 (32.6)	196 (54.4)	<.0001	23 (37.1)	29 (26.1)	.053
MVPA 40–<60 minutes/day	99 (30.3)	172 (39.3)	<.0001	81 (28.4)	108 (30)	<.0001	13 (21.0)	29 (26.1)	.053
MVPA ≥60 minutes/day	174 (53.2)	91 (20.8)	<.0001	111 (38.9)	56 (15.6)	<.0001	26 (41.9)	53 (47.7)	.053
**Maternal social economic status, n (%)**			.230	NA			NA		
Professional	17 (9.9)	13 (6.0)							
Managerial and technical	74 (43)	83 (38.4)							
Skilled nonmanual	49 (28.5)	76 (35.2)							
Skilled manual	<8 (1.2)	<8 (2.3)							
Partly skilled	25 (14.5)	31 (14.4)							
Unskilled	<8 (2.9)	8 (3.7)							
**Fasting plasma metabolic indices**	**15 years**			**17 years**			**24 years**		
High-density lipoprotein (mmol/L) mean (SD)	1.21 (0.27)	1.35 (0.28)	<.0001	1.18 (0.27)	1.33 (0.32)	<.0001	1.39 (0.35)	1.65 (0.41)	<.0001
Low-density lipoprotein (mmol/L) mean (SD)	2.00 (0.54)	1.65 (0.41)	<.0001	2.02 (0.56)	2.16 (0.54)	<.0001	2.49 (0.76)	2.43 (0.75)	.310
Triglyceride (mmol/L) median (IQR)	0.73 (0.34)	0.75 (0.40)	.106	0.76 (0.36)	0.73 (0.36)	.489	0.88 (0.56)	0.79 (0.42)	<.0001
Glucose (mmol/L) mean (SD)	5.30 (0.36)	5.14 (0.34)	<.0001	5.14 (0.36)	4.90 (0.33)	<.0001	5.49 (1.03)	5.21 (0.52)	<.0001
Hyperglycemia, n (%)	<8 (1.2)	<8 (0.2)	.169	<8 (1.5)	0	.034	24 (7.1)	17 (3.7)	.034
Insulin (mU/L) median (IQR)	8.09 (4.78)	9.74 (5.54)	<.0001	5.80 (4.31)	7.51 (4.14)	<.0001	7.38 (5.32)	7.68 (5.93)	.413
Hyperinsulinemia, n (%)	64 (19.0)	140 (30.8)	<.0001	37 (13.9)	47 (13.4)	.906	69 (20.5)	102 (22.4)	.542
HOMA-IR	1.91 (1.19)	2.21 (1.33)	<.0001	1.34 (1.06)	1.63 (1.01)	.011	1.86 (1.38)	1.77 (1.48)	.713
High sensitivity C-reactive protein (mg/L) Median (IQR)	0.34 (0.60)	0.34 (0.59)	.240	0.45 (0.59)	0.60 (1.30)	.005	0.64 (1.41)	1.00 (1.95)	<.0001

The values are means (SD) and median (interquartile range), except for lifestyle factors and ethnicity. Differences between sexes were tested using Student's t-test for normally distributed continuous variables, Mann–Whitney U test for skewed continuous variables, chi-square test for dichotomous variables, and analysis of covariance for multicategory variables. A 2-sided *P* < .05 is considered statistically significant. *P* value for sex differences. Hyperglycemia was categorized as fasting glucose >6.1 mmol/L, hyperinsulinemia was categorized as fasting insulin >11.28 mU/L.

Abbreviations: BMI, body mass index; HOMA-IR, homeostatic model assessment for insulin resistance; MVPA, moderate to vigorous physical activity; NA, not available/applicable.

### Mediating or Suppressing Effects of Body Composition and Lipids in the Longitudinal Associations of ST, LPA, and MVPA With Metabolic Indices

**Figure 2.**
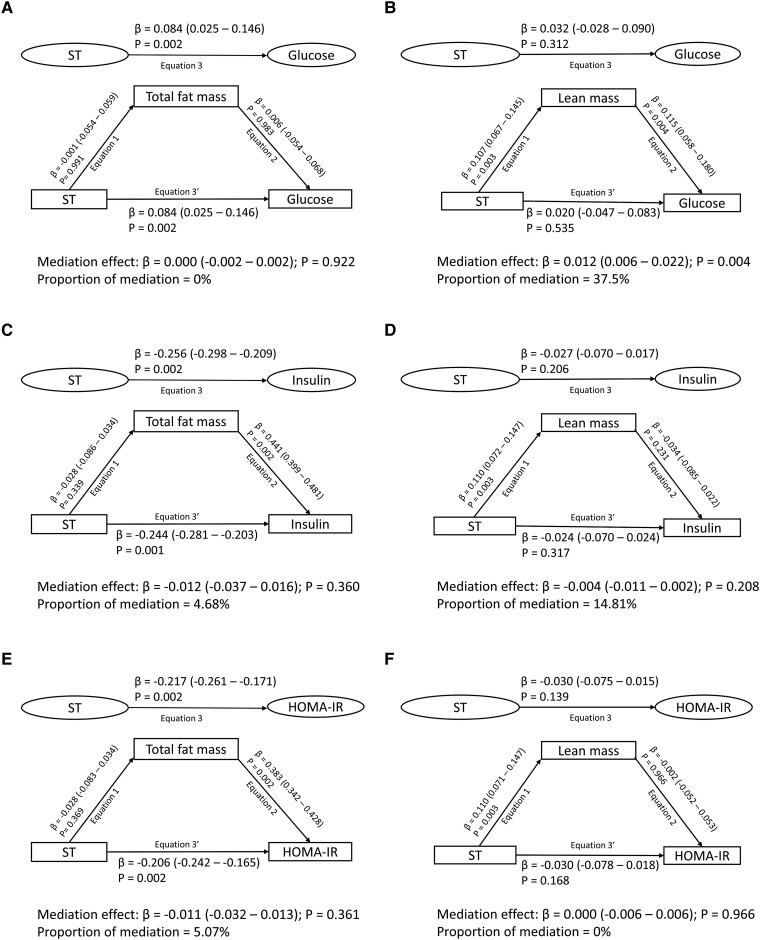
Mediating effect of cumulative fat mass and lean mass in the longitudinal associations of sedentary time (ST) with fasting plasma glucose, insulin and insulin resistance in 792 participants. (A) Sedentary time with glucose and total fat mass as a mediator. (B) Sedentary time with glucose and lean mass as a mediator. (C) Sedentary time with insulin and total fat mass as a mediator. (D) Sedentary time with insulin and lean mass as a mediator. (E) Sedentary time with insulin resistance and total fat mass as a mediator. (F) Sedentary time with insulin resistance and lean mass as a mediator. When the magnitude of the longitudinal association between the predictor and outcome is increased upon inclusion of a third variable, a suppression is confirmed; however, when decreased it is mediation. Mediation structural equation model estimating natural direct and indirect effects was adjusted for sex, family history of hypertension/diabetes/high cholesterol/vascular disease, and socioeconomic status, in addition to time-varying covariates such as age, high-sensitivity C-reactive protein, heart rate, systolic blood pressure, smoking status, light physical activity, moderate to vigorous physical activity, high-density lipoprotein cholesterol, low-density lipoprotein cholesterol, triglyceride, total fat mass, lean mass, glucose or insulin depending on the mediator and outcome. β is standardized regression coefficient. Two-sided *P* < .05 were considered statistically significant. HOMA-IR, homeostatic model assessment for insulin resistance.

**Figure 3.**
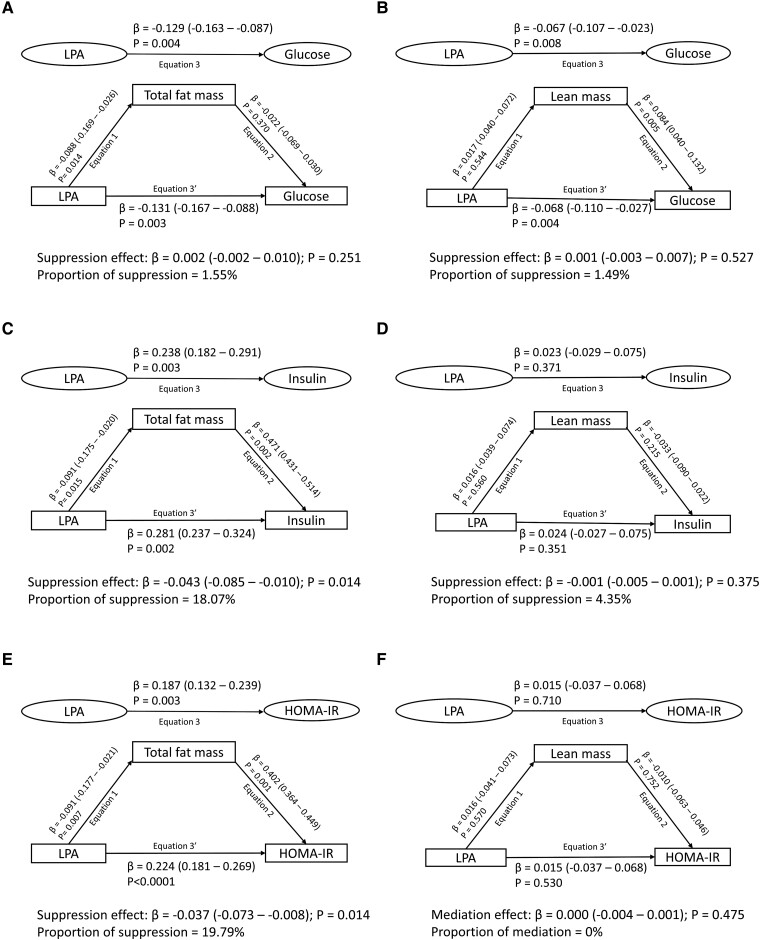
Mediating effect of cumulative fat mass and lean mass in the longitudinal associations of light physical activity (LPA) with fasting plasma glucose, insulin and insulin resistance in 792 participants. (A) Light physical activity with glucose and total fat mass as a mediator. (B) Light physical activity with glucose and lean mass as a mediator. (C) Light physical activity with insulin and total fat mass as a mediator. (D) Light physical activity with insulin and lean mass as a mediator. (E) Light physical activity with insulin resistance and total fat mass as a mediator. (F) Light physical activity with insulin resistance and lean mass as a mediator. When the magnitude of the longitudinal association between the predictor and outcome is increased upon inclusion of a third variable, a suppression is confirmed, but when decreased it is mediation. Mediation structural equation model estimating natural direct and indirect effects was adjusted for sex, family history of hypertension/diabetes/high cholesterol/vascular disease, and socioeconomic status, in addition to time-varying covariates such as age, high-sensitivity C-reactive protein, heart rate, systolic blood pressure, smoking status, sedentary time, moderate to vigorous physical activity, high-density lipoprotein cholesterol, low-density lipoprotein cholesterol, triglyceride, total fat mass, lean mass, glucose or insulin depending on the mediator and outcome. β is standardized regression coefficient. Two-sided *P* < .05 were considered statistically significant. HOMA-IR, homeostatic model assessment for insulin resistance.

Cumulative fat mass, lean mass, lipids, and inflammation had no significant mediating effect on the associations of cumulative ST with cumulative glucose, insulin, and HOMA-IR (Fig. 2; Table S2 (16)). Cumulative fat mass had partial suppression (18-20%) effects on the associations of cumulative LPA with cumulative insulin and HOMA-IR but not glucose (Fig. 3; Table S3 (16)). Cumulative lean mass, lipids, and inflammation had no significant mediating effect on the associations of cumulative LPA with cumulative glucose, insulin, and HOMA-IR (Fig. 3; Table S3 (16)).

**Figure 4.**
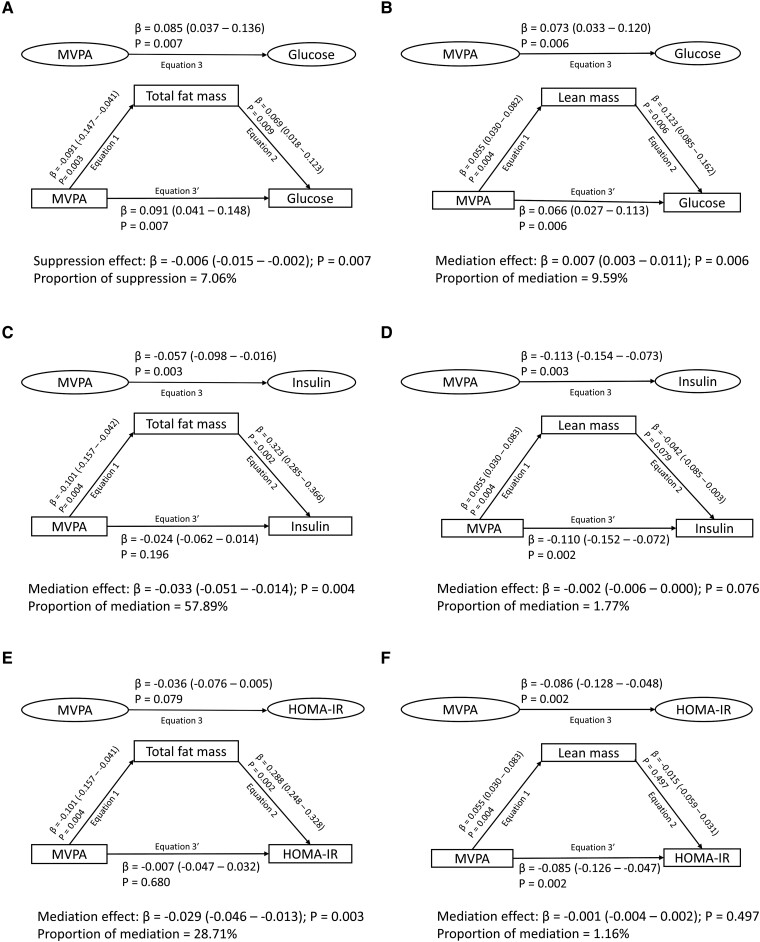
Mediating effect of cumulative fat mass and lean mass in the longitudinal associations of moderate to vigorous physical activity (MVPA) with fasting plasma glucose, insulin, and insulin resistance in 792 participants. (A) Moderate to vigorous physical activity with glucose and total fat mass as a mediator. (B) Moderate to vigorous physical activity with glucose and lean mass as a mediator. (C) Moderate to vigorous physical activity with insulin and total fat mass as a mediator. (D) Moderate to vigorous physical activity with insulin and lean mass as a mediator. (E) Moderate to vigorous physical activity with insulin resistance and total fat mass as a mediator. (F) Moderate to vigorous physical activity with insulin resistance and lean mass as a mediator. When the magnitude of the longitudinal association between the predictor and outcome is increased upon inclusion of a third variable, a suppression is confirmed, but when decreased it is mediation. Mediation structural equation model estimating natural direct and indirect effects was adjusted for sex, family history of hypertension/diabetes/high cholesterol/vascular disease, and socioeconomic status, in addition to time-varying covariates such as age, high-sensitivity C-reactive protein, heart rate, systolic blood pressure, smoking status, sedentary time, light physical activity, high-density lipoprotein cholesterol, low-density lipoprotein cholesterol, triglyceride, total fat mass, lean mass, glucose, or insulin depending on the mediator and outcome. β is standardized regression coefficient. Two-sided *P* < .05 were considered statistically significant. HOMA-IR, homeostatic model assessment for insulin resistance.

Cumulative total fat mass had partial suppression (7%) effect on the associations of cumulative MVPA with cumulative glucose but had strong mediating (58% and 29%) effects on the direct associations of cumulative MVPA with cumulative insulin and HOMA-IR, respectively (Fig. 4). Cumulative lean mass had a partial mediating (10%) effect on the associations of cumulative MVPA with cumulative glucose but none with insulin and HOMA-IR (Fig. 4). Cumulative high-density lipoprotein cholesterol had partial mediating (13-16%) effects on the association of cumulative MVPA with cumulative insulin and HOMA-IR, but not glucose (Table S4 (16)). Other lipids and inflammation had no mediation role in the association of MVPA with glucose, insulin, and HOMA-IR.

### Temporal Causal (Cross-lagged) and Inter-relational (Autoregressive) Associations of ST, LPA, and MVPA With Metabolic Indices

MVPA, glucose, insulin, and HOMA-IR at age 15 years were directly associated with their individual variables at age 24 years; however, ST at age 15 years was inversely associated with ST at age 24 years in the total cohort and among participants with overweight and obesity (Table 2; Table S5 (16)). LPA at 15 years was directly associated with LPA at 24 years in participants with overweight and obesity but not in the total cohort (Table 2; Table S5 (16)). There were no temporal or bidirectional relationships between movement behaviors and metabolic indices at ages 15-24 years observation period in the total cohort (Table S5 (16)).

**Table 2.** Autoregressive cross-lagged temporal causal longitudinal analyses of sedentary time and physical activity with fasting plasma glucose and insulin and insulin resistance at 15 and 24 years of age in 290 participants with overweight and obesity categorized using BMI ≥25 kg/m^2^ at age 24 years

	B	β	SE	*P*
Fasting glucose				
Autoregressive				
ST T1 —› ST T2	−0.361	−0.249	0.145	.013
LPA T1 —› LPA T2	0.069	0.098	0.012	<.0001
MVPA T1 —› MVPA T2	0.425	0.300	0.160	.008
Glucose T1 —› Glucose T2	0.472	0.340	0.087	<.0001
Cross-lagged				
ST T1 —› Glucose T2	0.000	0.035	0.000	.579
Glucose T1 —› ST T2	−23.058	−0.077	29.923	.441
LPA T1 —› Glucose T2	0.000	−0.017	0.001	.780
Glucose T1 —› LPA T2	−8.267	−0.075	1.967	<.0001
MVPA T1 —› Glucose T2	−0.001	−0.037	0.001	.548
Glucose T1 —› MVPA T2	−21.456	−0.245	9.933	.031
Fasting insulin				
Autoregressive				
ST T1 —› ST T2	−0.361	−0.249	0.145	.013
LPA T1 —› LPA T2	0.069	0.098	0.012	<.0001
MVPA T1 —› MVPA T2	0.425	0.300	0.160	.008
Insulin T1 —› Insulin T2	0.264	0.217	0.089	.003
Cross-lagged				
ST T1 —› Insulin T2	0.000	0.057	0.000	.358
Insulin T1 —› ST T2	−16.937	−0.031	63.913	.791
LPA T1 —› Insulin T2	−0.001	−0.165	0.000	.007
Insulin T1 —› LPA T2	−50.705	−0.257	4.202	<.0001
MVPA T1 —› Insulin T2	0.000	−0.003	0.001	.958
Insulin T1 —› MVPA T2	4.614	0.029	21.217	.828
HOMA-IR				
Autoregressive				
ST T1 —› ST T2	−0.386	−0.269	0.146	.008
LPA T1 —› LPA T2	0.072	0.102	0.014	<.0001
MVPA T1 —› MVPA T2	0.364	0.267	0.165	.027
HOMA-IR T1 —› HOMA-IR T2	0.077	0.061	0.082	.350
Cross-lagged				
ST T1 —› HOMA-IR T2	0.001	0.031	0.002	.627
HOMA-IR T1 —› ST T2	−3.393	−0.066	5.371	.528
LPA T1 —› HOMA-IR T2	−4.285	−0.178	0.402	.004
HOMA-IR T1 —› LPA T2	−0.008	−0.228	0.003	<.0001
MVPA T1 —› HOMA-IR T2	0.016	0.130	0.007	.037
HOMA-IR T1 —› MVPA T2	−1.183	−0.082	1.830	.518

Model was adjusted for baseline age, sex, low-density lipoprotein cholesterol, insulin, triglyceride, high sensitivity C-reactive protein, high-density lipoprotein cholesterol, heart rate, systolic blood pressure, glucose, fat mass, lean mass, smoking status, family history of hypertension/diabetes/high cholesterol/vascular disease, socioeconomic status, and insulin or glucose depending on outcome with additional adjustment for sedentary time (ST), light physical activity (LPA) or moderate to vigorous physical activity (MVPA) depending on the predictor. HOMA-IR, homeostatic model assessment for insulin resistance. Skewed variables were logarithmically transformed before analyses. A 2-sided *P* value <.05 is considered to be statistically significant.

Abbreviations: Time T1, 15 years of age; Time T2, 24 years; B, unstandardized regression; β, standardized regression, SE, standard error.

Among participants with overweight and obesity, ST had no temporal or bidirectional associations with glucose at the age 15- to 24-year observation period. Higher glucose, insulin, and HOMA-IR at age 15 years were associated with lower LPA at age 24 years, but higher LPA at age 15 years was associated with lower insulin and HOMA-IR but not glucose at age 24 years (Table 2). Higher glucose, insulin, and HOMA-IR at 15 years were associated with lower LPA at 24 years, but higher LPA at 15 years was associated with lower insulin and HOMA-IR but not glucose at 24 years (Table 2). Higher glucose but not insulin and HOMA-IR at 15 years was associated with lower MVPA at 24 years, while higher MVPA at 15 years was associated with higher HOMA-IR but not glucose and insulin at 24 years (Table 2).

### Longitudinal Associations of ST, LPA, and MVPA With Metabolic Indices

In the total cohort, cumulative ST from age 11-24 years was associated with decreased insulin and HOMA-IR but not glucose cumulatively measured from age 15-24 years, after full adjustments for cardiometabolic and lifestyle factors including LPA and MVPA (Table S6) (16). Cumulative LPA was associated with increased insulin and HOMA-IR but not glucose in the total cohort (Table S6 (16)). Cumulative MVPA and persistent MVPA of ≥60 minutes/day were not associated with metabolic indices (Table S6 (16)).

In participants with overweight and obesity higher cumulative ST was associated with an increased risk of progressively worsening hyperinsulinemia (odds ratio 1.20, 95% CI 1.00-1.44, *P* = .047) but not hyperglycemia and high HOMA-IR progression (Table S7 (16)). Higher cumulative LPA was associated with a decreased risk of progressive hyperinsulinemia (0.80, 0.66-0.96, *P* = .017) but not hyperglycemia and high HOMA-IR (Table S7 (16)). Cumulative MVPA and persistent MVPA of ≥60 minutes/day were not associated with the risk of poor metabolic indices (Table S7 (16)). In the total female cohort, cumulative ST was associated with decreased HOMA-IR, while cumulative LPA was associated with increased insulin and HOMA-IR but not glucose (Table S6 (16)). Movement behaviors were not associated with metabolic indices in males (Table S6 (16)).

## Discussion

This is the largest and longest follow-up study of children until young adulthood with objectively measured movement behaviors and metabolic indices which extends current evidence that movement behavior may be independently associated with poor metabolic indices, especially in the young population with overweight and obesity. This was concluded because, first, in the confounding longitudinal analyses, it was observed that cumulative ST was directly associated with an increased risk of hyperinsulinemia progression whereas cumulative LPA decreased the risk by the same amount in participants with overweight and obesity. Next, in the mediation analyses, it was observed that cumulative fat mass either mediated or suppressed the longitudinal relationships of either cumulative LPA or MVPA with glucose, insulin, and HOMA-IR in the total cohort. Lastly, from the temporal path analyses, it was observed that higher glucose in midadolescence temporally preceded lower LPA and MVPA in young adulthood, but higher insulin and HOMA-IR had bidirectional relationships with low LPA. Paradoxically, higher MVPA in midadolescence temporally preceded higher HOMA-IR in young adulthood.

### Sedentary Time and Metabolic Indices

The relationship between accelerometer-measured ST and metabolic indices may be determined by the obesity status of the pediatric population (13, 45). Increased ST may be less closely associated with metabolic disease risk, especially dysglycemia, in children than has been reported in adults (45, 46). In the total cohort, increased ST was inversely associated with insulin and HOMA-IR, but increased ST was positively associated with hyperinsulinemia in participants with overweight and obesity independent of LPA and MVPA. The finding in children with overweight and obesity over a 13-year follow-up is consistent with a previous report in children with overweight and obesity where objectively measured ST was associated with increased insulin secretion over a 7-year follow-up period (5). There were no mediating effects of traditional risk factors such as lipids, body composition, and inflammation in the relationship of ST with metabolic indices and lack of evidence of potential causal relationships (12, 14, 47). This likely suggests that the contribution of ST to metabolic derangement may be more complex in the pediatric population than earlier reported in experimental studies (45, 46, 48). ST may decrease the expression of anti-inflammatory and antioxidative modulators such as nicotinamide N-methyltransferase as well as regulators of glucose transporter type 4 translocation (46, 48). ST may also worsen arterial stiffness via hemodynamic vascular signaling dysregulation and development of inflammatory mediated atherogenesis which results in a poor metabolic profile but further studies are warranted (28, 43, 46, 49).

A recent study reported that cumulative ST was associated with higher fasting glucose in 9-year-olds with overweight and obesity followed up for 7 years, contrary to the present findings where ST was not associated with glucose in the total cohort and among children with overweight and obesity (5). This contrast may be partly due to (1) not accounting for muscle mass in the previous analyses, which is known to play an integral role in glucose homeostasis, (2) longer ST at midadolescence of 10.5 hours, and (3) having significant attrition with 377 of 630 baseline participants available at follow-up, where those lost to follow-up had significantly worse metabolic profiles (5). In the present study with shorter ST of 8 hours in midadolescence and a larger sample size of 792 participants at follow-up, lean muscle mass had ∼40% mediating role in the nonsignificant relationships of ST with glucose, and controlling for lean mass might have significantly attenuated any association. A large-scale longitudinal study recently reported that every 1 minute/day spent in ST was associated with a 1.3-gram increase in total fat mass and that both male and female children gained approximately 10 kilograms of fat mass, implying that ST contributed 700 grams to 1 kilogram of fat mass (approximately 7-10%) of the total fat mass gained during growth from childhood until young adulthood (13). This is clinically significant since a 1-kilogram fat mass increase among adults has been associated with a 20% to 50% increased risk of premature mortality (50). Similarly, cumulative ST contributed 67% of the increase in total cholesterol during growth from midadolescence to young adulthood (+0.46 mmol/L out of +0.69 mmol/L) (14). Increased lipids have been associated with subclinical atherosclerosis and a 20% to 30% increased risk of cardiac damage in youth (24, 47). Taken together, increased ST potentially causes increased total body fat and abdominal fat, and the later induces worsening fat mass–insulin resistance vicious cycle in the pediatric and young adult population, therefore ST may be considered an indirect risk factor for metabolic diseases (6, 13).

### Light Physical Activity and Metabolic Indices

Longitudinal evidence on objective measures of LPA in association with metabolic indices is scarce in the pediatric population partly due to the bias of multicollinearity between LPA, MVPA, and ST variables (2, 3, 5, 6, 10, 13, 14). In the present study, ST had a high correlation with LPA, which is consistent with a previous report (5); however, employing advanced statistical techniques better handled high correlations (13, 51). Among participants with overweight and obesity, higher cumulative LPA was associated with 20% decreased odds of progressive hyperinsulinemia, even after accounting for ST and MVPA and this relationship was partly suppressed by fat mass. Among adolescents, increased fat mass independently drives an increase in insulin thus, when fat mass was employed as a mediator it appears to contribute to a further increase in insulin (6). Among adults, LPA may yield metabolic health effects but with a 2- to 4-fold smaller effect on metabolic risks when compared to the same time spent in MVPA (52).

In the pediatric population, of the different movement behaviors, LPA may be the most optimal behavior for metabolic disease risk intervention, but little is known of its effectiveness (2, 5, 10, 53). However, emerging longitudinal studies have shown the beneficial effects of 3 to 4 hours/day of LPA in decreasing fat mass, inflammation, and cholesterol levels, which might help interrupt the vicious cycle of higher fat mass and insulin resistance in late adolescence (6, 12-14). Specifically, each 1 minute/day spent in LPA was associated with a 3.6-gram reduction in total fat mass implying that cumulative LPA decreased total body fat mass by 950 grams to 1.5 kilograms during growth from childhood to young adulthood, approximating 9.5% to 15% decrease in overall 10 kilograms gained in fat mass during the 13-year observation period (13). Moreover, higher cumulative LPA from childhood decreased total cholesterol by −0.53 mmol/L out of 0.69 mmol/L gained in total cholesterol during the growth from adolescence through young adulthood (approximately a 70% LPA-induced decrease in total cholesterol) (14). Thus, LPA might be a pragmatic target for future interventions and public health guidelines in the pediatric population since it is more feasible and accessible, requires less motivation, incidental to daily living, and does not require a high level of exercise skill or prior fitness (5, 52, 53).

### Moderate to Vigorous Physical Activity and Metabolic Indices

Longitudinal studies in children have reported that objective measures of MVPA were inversely associated with HOMA-IR, higher peripheral insulin sensitivity until midadolescence (3-5). However, the present study did not report any associations between cumulative MVPA and cumulative insulin or HOMA-IR from childhood through young adulthood, irrespective of sex and obesity status. This may partly be due to an ∼60% mediating effect of total fat mass which confirms previous knowledge that body fat has a significant detrimental effect on insulin sensitivity (6, 13, 54). Moreover, in the present study mean fasting insulin and HOMA-IR concentrations decreased between ages 15 and 17 years but increased between ages 17 and 24 years, while total fat mass significantly increased from ages 11 through 24 years. Consistent with the present finding, a previous longitudinal study in 9-year-olds concluded that MVPA attenuated the midadolescent peak in HOMA-IR, but by late adolescence the effect was lost (4). Paradoxically, it was observed that higher MVPA in mid-adolescence was associated with higher HOMA-IR in young adulthood in the present study. It is now established that each 1 minute/day spent in MVPA was associated with a 1.3-gram reduction in total fat mass resulting in a 70 to 170 gram (approximately 0.7-1.7%) reduction in 10 kilograms of total body fat mass gained during growth from childhood through young adulthood (13). Likewise, cumulative MVPA from childhood reduced total cholesterol by −0.05 mmol/L of the 0.69 mmol/L increase in total cholesterol (approximately a 7% MVPA-induced decrease in total cholesterol) during growth from midadolescence to young adulthood, but fat mass decreased the effect of MVPA on total cholesterol by nearly half (14). The susceptibility of MVPA to the dampening influence of fat mass is a novel discovery, which appears consistent and warrants further experimental and mechanistic explanations (12, 14). Taken together, the benefits of MVPA on HOMA-IR and insulin sensitivity seem restricted to childhood and midadolescence, and recommendations on PA intervention may consider LPA after late adolescence (12-14). Recently, movement behavior experts highlighted the public health importance of LPA in the young and adult population based on emerging evidence (53).

### Strength and Limitation

The extensive array of gold-standard and repeated measures of movement behavior, body composition, and covariates throughout the follow-up period in the ALSPAC data set offered the possibility of using advanced statistical models, to test the likelihood of reverse causality, temporality, potential causal explanatory pathway, and consistency of the findings. “The autoregressive cross-lagged temporal path analyses examine the effect of 1 variable on another variable at a later time-point. The cross-lagged effects are adjusted for the effect of each variable at 1 time on the same variable later, which is the autoregressive effect, which represents the stability of each variable over time (55). Both cross-lagged and autoregressive effects are analyzed simultaneously allowing for examining temporal precedence (55). Temporality assessed with cross-lagged analysis, which is a criterion for causal inference, is superior to cross-sectional correlational analysis (55). Moreover, the within-person level analyses may reflect potential causal effects than between-person associations (55). Observed or unobserved variables that are stable over time cannot, by design, confound within-level variables resulting in zero variations and correlations (55). Thus, significantly reducing the risk for confounding and eliminating potential confounders bias.” (14) The findings extend current evidence from adolescence to young adulthood and fill knowledge gaps that might be useful in updating future PA guidelines (2, 5, 10, 11). A few limitations are that the study participants were mostly White, thus generalization of findings to other racial and ethnic groups is limited. Moreover, residual confounding such as the unavailability of fasting metabolic indices at age 11 years, could bias the findings, however, metabolic measures at age 17 years were included in the analysis. Dietary variables were unavailable, but lipid profile and body composition were accounted for in the analysis. Objectively measured sleep data were not available, thus residual bias may not be excluded. Cohort attrition could lead to bias in observational studies, which may be negligible since participants who lacked certain movement behaviors and metabolic variables had similar characteristics to those included in the analyses. Accounting for age at pubertal maturation did not significantly alter the results. We did not measure insulin sensitivity and secretion using gold-standard methods such as the clamp test (56), given that the feasibility of these measures in large epidemiologic studies is limited; nonetheless, we used measures previously validated in the young population.

## Conclusion

Increased cumulative LPA strongly associates with a lower risk of hyperinsulinemia and insulin resistance progression from childhood through young adulthood. An increase in cumulative ST was associated with worsening metabolic indices, particularly in participants who were overweight and obese. Cumulative MVPA was not associated with metabolic indices but paradoxically associated with higher insulin resistance. The association of cumulative MVPA and metabolic indices may have been affected by the attenuating effect of increased fat mass. Promoting LPA while decreasing body fat mass and ST may be considered crucial intervention targets to attenuate the risk of dysglycemia, hyperinsulinemia, and insulin resistance from childhood through young adulthood.

## Data Availability

The informed consent obtained from ALSPAC participants does not allow the data to be made freely available through any third-party maintained public repository. However, data used for this submission can be made available on request to the ALSPAC Executive. The ALSPAC data management plan describes in detail the policy regarding data sharing, which is through a system of managed open access. Full instructions for applying for data access can be found here: http://www.bristol.ac.uk/alspac/researchers/access/. The ALSPAC study website contains details of all the data that are available (http://www.bristol.ac.uk/alspac/researchers/our-data/).

## Abbreviations

BMI: body mass index
GLMM: generalized linear mixed-effect model
HOMA-IR: homeostatic model assessment for insulin resistance
LPA: light physical activity
MVPA: moderate to vigorous physical activity
ST: sedentary time
